# Teacher interpersonal behaviors and student engagement in single-gender physical education: the mediating role of achievement emotions and the moderating effect of class gender composition

**DOI:** 10.3389/fpsyg.2026.1852105

**Published:** 2026-05-11

**Authors:** Yingkai Ma, Jihui Li

**Affiliations:** School of Physical Education, Shenyang Sport University, Shenyang, Liaoning, China

**Keywords:** achievement emotions, class engagement, moderated mediation, self-determination theory, single-gender physical education

## Abstract

Adolescent physical inactivity remains a prominent global public health challenge, and school physical education (PE) is the core setting to foster adolescents’ regular physical activity. While single-gender grouping is widely adopted in secondary PE worldwide, the mechanisms linking teacher interpersonal behaviors to student class engagement in this context remain underexplored. Grounded in the integrated framework of Self-Determination Theory and Control-Value Theory of Achievement Emotions, this study constructed a moderated mediation model to examine the effects of teachers’ need-supportive and need-thwarting behaviors on student PE engagement, with achievement emotions as the mediator and class gender composition as the moderator. A cross-sectional survey was conducted with 332 Grade 11 Chinese high school students, with data analyzed via SPSS, Process macro, and AMOS. Key results are presented as follows: (1). Teachers’ need-supportive behaviors positively predicted student engagement and positive achievement emotions, while need-thwarting behaviors negatively predicted engagement and positively predicted negative achievement emotions. (2). Positive achievement emotions exerted a fully mediating effect, and negative achievement emotions played a partially mediating effect in the associations between teacher interpersonal behaviors and student PE class engagement. (3). Class gender composition significantly moderated the direct predictive path from need-supportive behaviors to engagement, with a stronger positive effect observed in all-female single-gender PE classes. (4). The emotional mediating mechanism between teacher behaviors and student engagement remained invariant across all-male and all-female single-gender class groups. This study enriches theoretical research on single-gender PE and provides evidence-based pedagogical implications for optimizing single-gender PE teaching practices.

## 1 Introduction

Adolescent physical inactivity has become a prominent global public health challenge, with sufficient regular physical activity closely linked to adolescents’ physical health, mental well-being, cognitive development, and academic performance (Cimenti et al., 2025). School-based physical education (PE), as the most standardized, equitable, and accessible setting for adolescents to engage in physical activity, plays an irreplaceable role in fostering lifelong physical activity habits and promoting holistic youth development (Hemingway et al., 2026), with empirical evidence confirming single-gender PE as a critical context to elevate girls’ physical activity levels and associated health benefits (Wallace et al., 2020). Student engagement in PE classes, which encompasses agentic, cognitive, behavioral, and emotional dimensions, is not only the core indicator of PE teaching effectiveness, but also a direct and stable predictor of students’ long-term physical activity adherence and overall learning outcomes (Abbott-Chapman et al., 2014). For this reason, exploring the key antecedents and internal influencing mechanisms of students’ PE class engagement has long been a core research topic in sports education and educational psychology.

Teachers’ interpersonal behaviors, as the most proximal and critical environmental factors shaping students’ classroom experiences, have been widely confirmed to play a pivotal role in driving students’ learning motivation and engagement (Bai and Guo, 2026). Grounded in self-determination theory (SDT), existing research has distinguished between two core types of teacher interpersonal behaviors: need-supportive and need-thwarting interpersonal behaviors (Howard et al., 2025). While a large body of work has verified the positive effects of need-supportive teaching on PE student engagement, most studies have paid insufficient attention to the negative impact of need-thwarting behaviors, and few have simultaneously incorporated both types of behaviors into the same analytical framework to explore their differential effects in specific PE contexts. Empirical evidence has further demonstrated that need-thwarting teaching behaviors exert significant, irreversible detrimental effects on student motivation even when paired with high levels of need-supportive practices (Burgueño et al., 2024), with existing studies on PE teachers’ motivational styles further highlighting the importance of distinguishing between supportive and controlling behaviors while still lacking exploration in single-gender contexts (Fierro-Suero et al., 2024).

As a core affective factor in the educational process, achievement emotions have been proven to be a key bridge linking classroom environmental factors and student learning behaviors, as systematically elaborated in the control-value theory of achievement emotions (CVTAE) (Zhang and Han, 2026b). Prior research in PE has established the critical interplay between student motivation and achievement emotions in shaping learning outcomes, yet few studies have integrated both constructs within a single analytical framework (Fierro-Suero et al., 2023). Existing studies have initially verified the mediating role of achievement emotions between teaching behaviors and student engagement in PE settings, but the specificity of this mediating mechanism in single-gender PE classrooms has not been sufficiently verified, with most relevant work concentrated in mixed-gender class contexts. A systematic review on PE engagement further confirms that achievement emotions are a key enabler of student participation, and notes that the field lacks research on contextual boundary conditions such as single-gender grouping (Cimenti et al., 2025).

Single-gender grouping in PE classes is a widely adopted teaching model in secondary schools both in China and internationally, with large-scale empirical evidence confirming its prevalence as a mainstream practice to address gender gaps in PE participation, and documenting significant gender- and context-specific variations in student preferences for such grouping arrangements (Wilkinson et al., 2025; Wilkinson and Penney, 2025). Notably, the research context of this study is a Chinese high school that implements single-gender grouping only in PE courses, while maintaining mixed-gender teaching for all other academic subjects. This forms a unique educational scenario distinct from full single-gender schools, which has not received sufficient attention in existing research (Gee and Cho, 2014). Previous studies on gender differences in PE have mostly focused on individual gender differences between boys and girls in mixed-gender classes, while ignoring the moderating effect of class gender composition as the structural single-gender class context itself on the relationship between teaching behaviors and student outcomes (Frühauf et al., 2022). Empirical evidence from individual and cooperative PE game contexts further confirms that both student gender and group gender composition serve as significant predictors of positive and negative emotional experiences, with notable gender disparities observed across different instructional settings (Lavega et al., 2017). Existing work has found that female students have higher sensitivity to teachers’ interpersonal behaviors and classroom emotional environment (Ma et al., 2025), but this characteristic has not been fully verified in single-gender PE classrooms, and the stability of the emotional mediating mechanism across single-gender class groups remains unclear. Meanwhile, existing comparative studies on single-gender and coeducational PE have focused on students’ situational interest and skill learning outcomes, with limited exploration of how interpersonal interaction processes in single-gender PE classrooms shape student class engagement, a core predictor of long-term physical activity adherence (Lentillon-Kaestner and Roure, 2019). Cross-disciplinary evidence has further confirmed that class gender composition directly shapes students’ sensitivity to teacher-student interactions and subsequent class engagement, with significant gendered differences in the motivational benefits of single-gender versus mixed-gender learning environments (Almasri et al., 2021).

To address the above research gaps, this study integrates SDT and CVTAE to construct a moderated mediation model, and systematically explores the influence mechanism of teachers’ need-supportive and need-thwarting interpersonal behaviors on students’ PE class engagement in the unique scenario of PE-only single-gender grouping. The theoretical contributions of this study are mainly reflected in three aspects: first, it simultaneously incorporates need-supportive and need-thwarting interpersonal behaviors, expanding the contextual application of SDT in single-gender PE settings, beyond prior mixed-gender focused research on classroom climate and engagement; second, it integrates two core theories to reveal the key emotional mediating mechanism between teachers’ interpersonal behaviors and student engagement, filling the gap of insufficient attention to emotional mechanisms in previous single-gender PE research; third, it clarifies the boundary conditions of the research model, verifying both the gender difference in the direct effect of supportive behaviors and the cross-group stability of the core emotional mediating mechanism. In practice, this study can provide targeted, evidence-based guidance for the optimization of teaching practices and differentiated instruction in single-gender PE classes.

## 2 Theoretical framework and hypotheses development

### 2.1 Core theoretical foundations

#### 2.1.1 Self-determination theory (SDT)

SDT is a classic macro theory of human motivation and personality development, focusing on the degree to which human behavior is self-determined and autonomous (Ryan et al., 2025). Its core proposition holds that humans have three innate, universal, cross-cultural basic psychological needs: autonomy, competence, and relatedness. Satisfaction of these needs drives optimal motivation, positive emotional experiences, and sustained positive behaviors, while need frustration leads to diminished motivation, negative affect, and maladaptive behaviors (Howard et al., 2025).

In educational contexts, teachers’ interpersonal behaviors are the most direct predictor of students’ basic psychological need satisfaction or frustration. Need-supportive interpersonal behaviors are operationalized along three intercorrelated dimensions: autonomy support, competence support, and relatedness support. Need-thwarting interpersonal behaviors encompass the corresponding dimensions of autonomy thwarting, competence thwarting, and relatedness thwarting. This six-factor structure has been rigorously validated with robust psychometric properties and cross-gender invariance in secondary school PE settings (Burgueño and Medina-Casaubón, 2021). Need-supportive behaviors uphold students’ autonomous choice, deliver targeted guidance to build competence, and foster warm, inclusive teacher-student relationships (Patzak and Zhang, 2025). These behaviors effectively satisfy students’ three basic psychological needs, thereby promoting positive learning experiences and active class engagement. Intervention research confirms that integrating need-supportive strategies into PE pedagogical models significantly enhances student motivation and engagement, highlighting the practical value of SDT in PE settings (Flores-Cidoncha et al., 2025). In contrast, need-thwarting interpersonal behaviors, including controlling, demeaning, and alienating actions, frustrate students’ basic psychological needs, induce negative emotional experiences, and drive behavioral withdrawal and reduced class participation (Shi et al., 2025). Extensive PE research has confirmed the differential predictive effects of these two behavior types on students’ PE experiences and participation, providing a solid theoretical foundation for this study.

Further research emphasizes the reciprocal dynamic between teachers’ motivating styles and student engagement: students’ in-class engagement can in turn shape teachers’ adoption of need-supportive or need-thwarting interpersonal behaviors, which is a core focus of PE motivation research (Van Doren et al., 2026). Cross-cultural studies have identified key antecedents of PE teachers’ adoption of need-supportive or need-thwarting motivating styles, providing contextual grounding for the examination of teacher behaviors in this study (Csordás-Makszin et al., 2025). Meanwhile, recent research among Dutch secondary students further validated the robust differential effects of need-supportive and need-thwarting interpersonal behaviors on students’ classroom experiences and engagement, enriching the cross-cultural generalizability of the SDT framework in educational settings (Villegas et al., 2026). Empirical evidence also verifies that teacher autonomy support, a core facet of need-supportive behaviors, directly and indirectly shapes students’ academic emotions, establishing a critical theoretical link between SDT and the control-value framework adopted in this study (Tong, 2025).

#### 2.1.2 Control-value theory of achievement emotions (CVTAE)

CVTAE is the core theoretical framework explaining the generation mechanism and functional role of achievement emotions in educational settings, systematically elaborating the antecedents, structure, and outcome effects of students’ achievement emotions (Zhang and Han, 2026b). The theory defines achievement emotions as emotional experiences directly tied to students’ academic achievement activities and outcomes, categorized by valence into positive achievement emotions (e.g., enjoyment, pride) and negative achievement emotions (e.g., anger, anxiety, hopelessness, boredom).

According to CVTAE, the classroom environment is the core antecedent of students’ achievement emotions, with teachers’ teaching behaviors and interpersonal interactions as its most critical components (Zhang and Han, 2026b). Research on de-motivating teaching approaches further confirms that teacher behaviors shape student achievement emotions through need-based experiences, providing empirical support for the CVTAE framework in PE contexts (Diloy-Peña et al., 2026). Regarding outcome variables, achievement emotions significantly predict students’ learning engagement and academic performance. Positive achievement emotions broaden students’ attentional scope, enhance learning initiative, and increase class participation, while negative achievement emotions consume cognitive resources, reduce learning interest, and drive class disengagement. Notably, specific negative achievement emotions (e.g., moderate task-related anxiety) may also facilitate adaptive learning behaviors (Wang and Zheng, 2023). Existing PE research has verified that PE teachers’ teaching behaviors significantly predict students’ in-class achievement emotions, which in turn shape their class engagement, providing a basis for exploring the mediating role of achievement emotions in this study.

#### 2.1.3 Theoretical integration

SDT and CVTAE exhibit strong theoretical complementarity, forming the systematic integrated research framework for this study. SDT reveals the essential attributes of teachers’ interpersonal behaviors, explaining why need-supportive and need-thwarting behaviors drive differential student psychological and behavioral outcomes via basic psychological need satisfaction and frustration. Critically, this need-based experience acts as the core motivational mechanism linking teacher behaviors to achievement emotions: need satisfaction enhances students’ perceived control and task value of PE (the core antecedents of achievement emotions in CVTAE), while need frustration diminishes these appraisals. CVTAE then supplements this framework by delineating the specific emotional transmission path, clarifying how these appraisals link teachers’ interpersonal behaviors, as a core classroom environmental factor, shape students’ achievement emotions and subsequent class engagement.

In the single-gender PE classroom context, this study proposes an integrated theoretical logic: teachers’ need-supportive interpersonal behaviors satisfy students’ basic psychological needs, enhance their PE task control and value appraisals, induce positive achievement emotions, and thereby promote their multi-dimensional class engagement. Conversely, teachers’ need-thwarting interpersonal behaviors frustrate students’ basic psychological needs, reduce their task control and value appraisals, trigger negative achievement emotions, and inhibit class participation. Meanwhile, class gender composition acts as a boundary condition, moderating the strength of the direct path, while the core emotional mediating mechanism may remain stable across groups. Furthermore, cross-cultural evidence verifying its robust mediating role between teacher behaviors and student learning outcomes (Ma et al., 2024). The following research hypotheses are proposed based on this integrated framework.

## 3 Hypotheses development

### 3.1 Main effect of teacher interpersonal behaviors (H1)

Consistent with SDT’s core propositions, extensive empirical research has confirmed teachers’ need-supportive interpersonal behaviors as key positive predictors of students’ learning engagement (Howard et al., 2025). In PE contexts, teachers’ autonomy support grants students independent choices in learning content and practice methods, stimulating autonomous motivation for PE participation (Llanos-Muñoz et al., 2025). Competence support delivers targeted skill guidance and positive feedback, enhancing students’ sports self-efficacy (Patzak and Zhang, 2025). Relatedness support builds equal, inclusive teacher-student relationships, helping students feel accepted and respected in PE classes (Koeppen et al., 2025). These need-supportive behaviors effectively satisfy students’ three basic psychological needs, significantly promoting their multi-dimensional class engagement in PE. A longitudinal study further confirms the bidirectional positive relationship between teacher support and student engagement in middle school PE, highlighting the robustness of this effect (Zhang and Han, 2026a).

In contrast, teachers’ need-thwarting interpersonal behaviors exert a significant negative impact on students’ PE class engagement. Teachers’ controlling behaviors deprive students of autonomy, demeaning language undermines their competence, and alienated attitudes frustrate their need for relatedness (Shi et al., 2025). These behaviors reduce students’ autonomous motivation for PE, induce negative emotional experiences, and lead to reduced in-class effort, practice avoidance, and even resistance to PE courses (Fierro-Suero et al., 2024). A meta-analysis also confirmed a stable negative correlation between teachers’ need-thwarting behaviors and students’ class engagement across educational contexts (Howard et al., 2025). Additionally, per CVTAE, teachers’ interpersonal behaviors, as a core classroom environmental factor, directly impact students’ achievement emotions (Zhang and Han, 2026b). The first hypothesis is therefore proposed: H1: In single-gender PE classes, teachers’ need-supportive interpersonal behaviors have a significant positive predictive effect on students’ class engagement and positive achievement emotions; teachers’ need-thwarting interpersonal behaviors have a significant negative predictive effect on students’ class engagement and a significant positive predictive effect on students’ negative achievement emotions.

### 3.2 Mediating effect of achievement emotions (H2)

Per the integrated SDT-CVTAE, achievement emotions are the key mediating mechanism linking teacher classroom environmental factors and students’ learning behaviors (Zhang and Han, 2026b). Teacher behaviors first shape students’ basic psychological need satisfaction and frustration, which alters their PE task control and value appraisals, and in turn drives changes in achievement emotions. This study proposes that teachers’ interpersonal behaviors are associated with students’ PE class engagement via their achievement emotions, forming a hypothesized significant mediating associational path.

For the positive path, teachers’ need-supportive interpersonal behaviors create a safe, inclusive classroom atmosphere, satisfy students’ basic psychological needs, and effectively stimulate positive achievement emotions such as enjoyment and pride in PE classes (Diloy-Peña et al., 2026). These positive emotions broaden students’ attentional scope, enhance learning initiative, promote deep cognitive processing, and significantly improve multi-dimensional class engagement (Daroglou et al., 2026). Existing research has found that positive achievement emotions play a significant mediating role between need-supportive teaching and students’ PE class participation (Zhang and Han, 2026b), with evidence from Chinese middle school PE settings further confirming this mediating effect, providing direct empirical support for this hypothesized pathway in the Chinese cultural context (Zhang et al., 2024).

For the negative path, teachers’ need-thwarting interpersonal behaviors frustrate students’ basic psychological needs, readily inducing negative achievement emotions including anger, anxiety, hopelessness, and boredom in PE classes (Shi et al., 2025). Anxiety directs excessive attention to negative evaluation and consumes cognitive resources; anger triggers resistance to classroom rules and activities; hopelessness reduces initiative to attempt challenging skills; boredom directly diminishes interest and initiative in PE learning. All four types of negative achievement emotions significantly inhibit students’ class engagement (Gu and Cheng, 2026). Previous studies have confirmed negative achievement emotions as key intermediary variables between controlling teaching and students’ learning withdrawal behaviors. Based on the above analysis, the second hypothesis is proposed: H2: In single-gender PE classes, positive achievement emotions play a significant positive mediating role between teachers’ need-supportive interpersonal behaviors and students’ class engagement; negative achievement emotions play a significant negative mediating role between teachers’ need-thwarting interpersonal behaviors and students’ class engagement.

### 3.3 Moderating effect of class gender composition on the positive direct path (H3)

Existing studies consistently document significant gender differences in students’ sensitivity to teachers’ interpersonal behaviors, with female students generally showing higher sensitivity to positive, supportive interpersonal interactions. Notably, this sensitivity is further shaped by the class gender composition context, rather than being solely determined by individual gender (Ma et al., 2025). PE-specific empirical evidence confirms that girls exhibit greater responsiveness to both need-supportive and need-thwarting teaching behaviors compared to boys, with gendered differences in the associations between teacher behaviors and student motivational outcomes well documented in secondary school PE contexts (Abós et al., 2022). In mixed-gender PE classes, girls face greater vulnerability to gender stereotypes and opposite-sex social pressure, while the all-female single-gender class environment eliminates these stressors, leading girls to prioritize the quality of supportive interpersonal interactions with teachers (Yun et al., 2026). In contrast, boys’ PE class engagement is more strongly driven by sports competition, skill improvement, and peer interaction, resulting in relatively lower sensitivity to teachers’ need-supportive behaviors than girls (Koh et al., 2019). Qualitative research also finds that girls prioritize teachers’ emotional support and respectful attitude in PE classes, while boys focus more on teachers’ professional competence and fairness (Fernández-Aránegas et al., 2025).

Notably, the PE-only single-gender grouping context eliminates the confounding effect of full single-gender school environments, enabling more precise examination of how class gender composition shapes students’ responses to teacher behaviors. Based on this evidence, the third hypothesis is proposed: H3: Class gender composition has a significant moderating effect on the direct predictive effect of teachers’ need-supportive interpersonal behaviors on PE class engagement: the positive predictive effect of need-supportive interpersonal behaviors on students’ class engagement is significantly stronger in all-female classes than in all-male classes.

### 3.4 Cross-group stability of the emotional mediating mechanism (H4)

While the aforementioned gender differences in sensitivity to teacher interpersonal behaviors are well documented, CVTAE posits that the core emotional mediating path linking classroom environmental factors to student learning behaviors holds cross-contextual universality (Zhang and Han, 2026b). The gendered differences in responsiveness to teacher behaviors documented in prior research (Abós et al., 2022; Ma et al., 2025) may manifest in the strength of the direct predictive path, rather than altering the fundamental sequential mechanism from teacher behaviors to achievement emotions, and in turn to class engagement. The PE-only single-gender grouping context, by eliminating mixed-gender stereotype pressures, may further equalize the core emotional processing mechanism across male and female student groups (Yun et al., 2026).

Building on this, the fourth hypothesis is proposed: H4: The mediating effect of achievement emotions between teachers’ interpersonal behaviors and PE class engagement is stable across all-male and all-female single-gender classes: there is no significant difference in the magnitude of the mediating effect between all-female and all-male classes.

## 4 Materials and methods

### 4.1 Study design and ethical approval

This study adopted a cross-sectional questionnaire survey design, which is suitable for exploring the correlation and predictive relationships between variables in the established theoretical model. The research protocol has been reviewed and formally approved by the institutional ethical review authority of the participating high school, in full compliance with the WMA Declaration of Helsinki and educational research ethical norms. A waiver of written informed consent from the legal guardians of minor participants was approved by the ethical review authority, based on the study’s fully anonymous design, no more than minimal risk to participants, and full disclosure of all study details to guardians through official school-home communication channels with no objections received during the public notification period. All surveys were conducted anonymously, with all participants fully informed of the research purpose, anonymity principle, voluntary participation rule, and data confidentiality commitment before the survey. Participants were explicitly notified that their participation was completely voluntary, they could withdraw from the survey at any time without any negative consequences, and all collected data would be kept strictly confidential and used exclusively for academic research purposes.

### 4.2 Participants

This study adopted convenience sampling to select Grade 11 students from a full-time general high school in China as participants. Grade 11 students were selected for two core reasons: first, they have formed stable PE participation patterns after 1 year of senior high school adaptation; second, they are not yet exposed to the extreme academic pressure of the national college entrance examination, ensuring the authenticity of responses reflecting daily PE experiences and avoiding response bias from college entrance examination-related stress. The school implements single-gender class grouping exclusively in physical education courses, while all other academic courses adopt mixed-gender class teaching, which fully conforms to the situational definition of single-gender PE classes in this study and avoids the confounding effect of full single-gender school environments (Gee and Cho, 2014). All participants are full-time high school students with no personal income, and have a homogeneous socioeconomic status (SES) background. This design effectively controls the potential confounding effect of SES on students’ PE engagement and teachers’ motivational teaching practices.

A total of 350 questionnaires were distributed in this study, including 175 for all-male single-gender PE classes and 175 for all-female single-gender PE classes. After invalid sample screening, 332 valid questionnaires were recovered, with an effective recovery rate of 94.86%. Among the valid samples, 170 are from students in all-male single-gender PE classes, and 162 are from students in all-female single-gender PE classes.

An *a priori* power analysis was conducted using G*Power 3.1.9.7 software to determine the minimum required sample size for the core linear multiple regression models. The analysis was set with a medium effect size f^2^ = 0.15, a two-tailed significance level *α* = 0.05, a statistical test power (1-*β*) = 0.95, and 6 predictors included in the regression model. The results showed that the minimum required total sample size was 146. The total valid sample (*N* = 332), the all-male subgroup sample (*n* = 170), and the all-female subgroup sample (*n* = 162) of this study are all well above this threshold, fully meeting the statistical requirements of all subsequent analyses.

### 4.3 Measures

All scales used in this study are mature, validated scales that have been widely used in previous SSCI studies and verified to have good reliability and validity in the Chinese adolescent PE context. All items are presented in simplified Chinese, with Likert scale scoring according to the original scale design.

### 4.4 Independent variables: teacher interpersonal behaviors

The Interpersonal Behaviors Questionnaire (IBQ) was used to measure students’ perceptions of teachers’ interpersonal behaviors in PE classes (Rocchi et al., 2017a). The scale includes two core dimensions: need-supportive interpersonal behaviors and need-thwarting interpersonal behaviors. Each dimension has 3 sub-dimensions (autonomy support/thwarting, competence support/thwarting, relatedness support/thwarting), with 4 items per sub-dimension, resulting in a total of 24 items for the full scale (12 items per dimension). A 7-point Likert scale was used for scoring, ranging from 1 = completely inconsistent to 7 = completely consistent. The mean score of the items in each dimension was calculated respectively, with a higher score indicating a higher level of the corresponding teacher interpersonal behavior perceived by students. The scale has been verified to have good cross-gender validity in sports contexts (Rocchi et al., 2017b). Example items: autonomy support (“My PE teacher gives me freedom to choose practice content”), competence thwarting (“My PE teacher makes me feel incompetent in sports”), relatedness support (“My PE teacher understands my feelings when I struggle with skills”). The full simplified Chinese version is in the Supplementary Material.

### 4.5 Mediating variables: PE class achievement emotions

The Achievement Emotions Questionnaire for Physical Education (AEQ-PE) was used to measure students’ achievement emotions in PE classes (Fierro-Suero et al., 2020). The scale includes two core dimensions: positive achievement emotions and negative achievement emotions. The positive dimension includes 2 sub-dimensions (pride, enjoyment) with 8 items in total; the negative dimension includes 4 sub-dimensions (anger, anxiety, hopelessness, boredom) with 16 items in total, resulting in 24 items for the full scale. A 5-point Likert scale was used for scoring, ranging from 1 = completely inconsistent to 5 = completely consistent. The mean score of the items in each core dimension was calculated respectively, with a higher score indicating a higher level of the corresponding achievement emotion. The scale has been verified to have good reliability and cross-gender stability in the Chinese adolescent PE context (Fierro-Suero et al., 2020). Example items for each dimension are as follows: positive achievement emotions (“I feel proud of my performance in PE class”), and negative achievement emotions (“I feel bored during PE activities”). The full simplified Chinese version of the scale is provided in the Supplementary Material.

### 4.6 Dependent variable: PE class engagement

The Engagement in Physical Education Scale (EPES) was used to measure students’ multi-dimensional engagement in PE classes (Stringfellow et al., 2024). The scale includes 4 sub-dimensions: agentic engagement, cognitive engagement, behavioral engagement, and emotional engagement, with a total of 18 items. A 5-point Likert scale was used for scoring, ranging from 1 = completely inconsistent to 5 = completely consistent. The mean score of the 18 items was calculated, with a higher score indicating a higher level of students’ PE class engagement. Example items for each dimension are as follows: behavioral engagement (“I try my hardest in PE class”), and agentic engagement (“I ask the teacher questions to help my learning when needed”). The full simplified Chinese version of the scale is provided in the Supplementary Material.

### 4.7 Moderating variable: class gender composition

This variable was measured through a single demographic question: “Your current PE teaching class is: 1=all-male single-gender class, 2=all-female single-gender class”. It is a binary nominal variable, which was recoded into a dummy variable for statistical analysis: 0 = all-male single-gender class, 1 = all-female single-gender class, with the all-male class set as the reference group.

### 4.8 Attention check items

Three attention check items were set to test the validity and seriousness of responses, with one item embedded at the beginning of each of the three core scales (before the formal items of each questionnaire) to avoid respondents’ advance detection. Each item required respondents to select a pre-specified response option (e.g., “Please select ‘Somewhat disagree’ for this question”) to verify attentive reading. Only participants who correctly answered all three items were retained in the final valid sample, eliminating invalid responses from inattentive participants. These items were used to screen invalid response samples and improve the overall quality of the collected data.

### 4.9 Data collection procedure

The questionnaire distribution was conducted by well-trained investigators during indoor PE theory classes. This dedicated, distraction-free classroom setting ensured a quiet, focused environment for students to complete the survey, supporting sustained attention throughout the process. First, the investigators explained the research purpose, anonymity principle, voluntary participation principle, and data confidentiality commitment to the students. After confirming the students’ informed consent, the questionnaires were distributed to the students. After completion, the questionnaires were uniformly collected by the PE class representatives of each class, and the course teachers did not have access to the questionnaire content throughout the process, to avoid students’ concerns about answering truthfully due to the presence of teachers. The estimated completion time of the questionnaire was about 15 min. After recovery, the investigators checked the integrity of the questionnaires on the spot, and screened out invalid samples according to pre-set rules.

### 4.10 Data processing and statistical analysis

All data processing and statistical analysis in this study were completed using IBM SPSS Statistics 27.0 software, SPSS Process macro, and AMOS 24.0, in strict accordance with APA 7th edition guidelines for quantitative research reporting. The specific analysis process is as follows: Data Preprocessing: First, invalid samples were screened out in accordance with the following rules: (1) Only samples with all three attention check items answered correctly were retained, and any sample with an incorrect answer to any of the attention check items was excluded; (2) Samples with uniform responses (i.e., selecting the same option for all scale items) were excluded; (3) Only complete questionnaires with no missing values across all scales were retained, and any sample with missing values was excluded.

Common Method Bias (CMB) Test: The combination of Harman’s single factor test and AMOS-based single-factor confirmatory factor analysis was used to test the CMB of this study.

Reliability and Validity Test: Cronbach’s *α* coefficient was used to test the internal consistency reliability of each scale; composite reliability (CR) and average variance extracted (AVE) were used to test the convergent validity of the scale; the comparison between the square root of AVE and the correlation coefficient between variables was used to test the discriminant validity. AMOS 24.0 was used to conduct confirmatory factor analysis (CFA) to verify the structural validity of the measurement model.

Descriptive Statistics and Correlation Analysis: The mean and standard deviation of each core variable were calculated, and the Pearson correlation coefficient between variables was analyzed.

Main Effect and Moderation Effect Test: Hierarchical regression analysis with HC3 heteroscedasticity-robust standard errors was used to test the main effect of teachers’ interpersonal behaviors on students’ class engagement and the moderating effect of class gender composition. AMOS-based structural equation modeling (SEM) was used to verify the robustness of the main effect path.

Mediation Effect Test: Model 4 of the Process macro was used, with 5,000 bias-corrected Bootstrap samples and 95% confidence interval, to test the hypothesized mediating associational pathway of positive or negative achievement emotions. This analytical approach is a well-established, widely adopted standard paradigm in sports psychology and educational research for examining mediation effects in questionnaire-based studies (Yiming et al., 2023). AMOS-based Bootstrap method was used to cross-validate the significance of the hypothesized mediating associational pathway.

Cross-Group Invariance Test of the Mediating Associational Pathway: Model 8 of the Process macro was used, with 5,000 bias-corrected Bootstrap samples and HC3 heteroscedasticity-robust standard errors, to test the between-group difference in the magnitude of the mediating associational effect. This moderated mediation analytical framework has been rigorously validated and widely applied in prior research on motivational processes in physical education contexts (Yiming et al., 2023). AMOS-based multi-group invariance test was used to verify the cross-group stability of the measurement model and structural mediating path.

Notably, the current study relied on student self-report measures to assess perceived teacher interpersonal behaviors, consistent with the dominant methodological approach in existing SDT-based PE research. While observational assessments of teaching behaviors represent an alternative methodological approach with complementary strengths (Teraoka et al., 2024), the self-report method was selected to capture students’ subjective perceptual experiences, which are the core proximal predictors of emotional and motivational outcomes aligned with the theoretical foundations of this study.

## 5 Results

### 5.1 Common method bias test

Harman’s single-factor test was performed to examine potential common method bias. The results showed that the first unrotated factor explained 28.03% of the total variance, well below the 40% threshold. Meanwhile, the AMOS-based single-factor CFA results showed poor model fit (*χ*^2^/df = 31.937, CFI = 0.262, TLI = 0.163, RMSEA = 0.306, SRMR = 0.311), which was significantly worse than the five-factor measurement model, confirming that common method bias is not a concern in this study.

### 5.2 Reliability and validity test results

To confirm the psychometric properties of the core measurement scales, reliability and convergent validity tests were conducted for all latent dimensions. The results are presented in Table 1. All Cronbach’s *α* coefficients of the core dimensions were above 0.80, indicating excellent internal consistency reliability. All composite reliability (CR) values were above 0.70, and all average variance extracted (AVE) values were above 0.50, indicating ideal convergent validity (Zhang and Jin, 2025).

**Table 1:** Reliability and validity test results of the scales.

Dimension	Number of items	Cronbach’s *α*	CR	AVE
Need-supportive interpersonal behaviors	12	0.935	0.909	0.768
Need-thwarting interpersonal behaviors	12	0.900	0.794	0.563
Positive achievement emotions	8	0.888	0.865	0.763
Negative achievement emotions	16	0.927	0.843	0.578
PE class engagement	18	0.928	0.902	0.696

CR, Composite Reliability; AVE, Average Variance Extracted. All Cronbach’s α coefficients exceed the 0.70 threshold for acceptable internal consistency reliability, and all CR values exceed the 0.70 threshold for good composite reliability. All AVE values are above the 0.50 threshold for ideal convergent validity.

**Figure 1:**
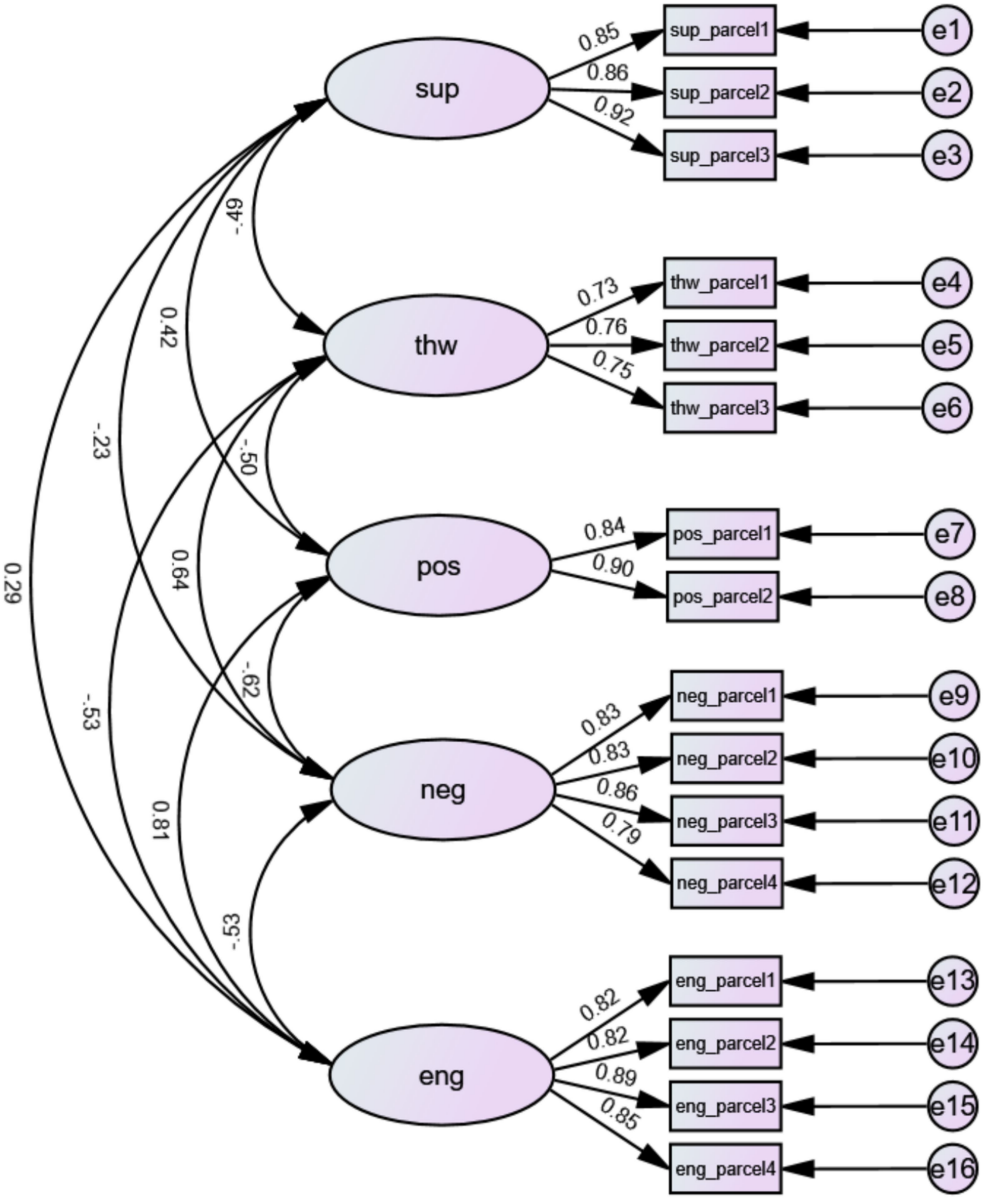
Standardized path coefficients of the latent variable structural equation model (measurement model and structural model). Sup, Need-supportive Interpersonal Behaviors; Thw, Need-thwarting Interpersonal Behaviors; Pos, Positive Achievement Emotions; Neg, Negative Achievement Emotions; Eng, PE Class Engagement. The values on the arrows are standardized factor loadings and path coefficients; all factor loadings are statistically significant at *p* < 0.001 level.

Confirmatory factor analysis (CFA) was conducted via AMOS 24.0 to examine the structural validity of the five-factor measurement model (need-supportive interpersonal behaviors, need-thwarting interpersonal behaviors, positive achievement emotions, negative achievement emotions, and PE class engagement). The model fit indices were as follows: *χ*^2^ = 311.076, df = 94, *χ*^2^/df = 3.309, CFI = 0.942, TLI = 0.925, RMSEA = 0.108, SRMR = 0.038, GFI = 0.887, AGFI = 0.836, indicating that the measurement model had acceptable structural validity and good fit to the data. The final structural equation model with standardized path coefficients is presented in Figure 1. Separate confirmatory factor analyses were conducted for each full scale and its core dimensions. Fit indices are presented in Table 2.

**Table 2:** Fit indices of confirmatory factor analyses for each scale.

Scale	*χ*^2^	df	*χ*^2^/df	CFI	TLI	RMSEA (90%CI)	SRMR
Interpersonal behaviors questionnaire (IBQ, 2-factor)	56.824	8	7.103	0.954	0.914	0.136 (0.104–0.170)	0.059
Need-supportive dimension	0.000	0	–	1.000	1.000	0.000	0.000
Need-thwarting dimension	0.000	0	–	1.000	1.000	0.000	0.000
Achievement emotions questionnaire for PE (AEQ-PE, 2-factor)	81.404	8	10.175	0.942	0.891	0.166 (0.135–0.200)	0.024
Positive achievement emotions	0.000	0	–	1.000	1.000	0.000	0.000
Negative achievement emotions	47.892	2	23.945	0.945	0.835	0.263 (0.202–0.330)	0.025
Engagement in physical education scale (EPES)	29.500	2	14.750	0.970	0.909	0.204 (0.143–0.272)	0.026

Subdimensions with only 3 parcel indicators were just-identified (df = 0), resulting in mathematically perfect fit. Models with extremely small degrees of freedom tend to inflate RMSEA and *χ*^2^/df values. All core incremental fit indices (CFI, TLI, SRMR).

Multi-group measurement invariance across gender was examined, with results presented in Table 3.

**Table 3:** Multi-group measurement invariance test results across gender.

Invariance model	*χ*^2^	df	Δ*χ*^2^	Δdf	ΔCFI	ΔRMSEA	Invariance established
Configural invariance	471.262	188	–	–	–	–	–
Metric invariance	471.262	188	0.000	0	0.000	0.000	Yes
Scalar invariance	588.839	203	117.577	15	−0.028	0.008	Partial

Cut-off criteria for invariance: ΔCFI ≤ − 0.01, ΔRMSEA ≤ 0.015. Partial scalar invariance is supported for subsequent cross-group comparisons, consistent with established SEM reporting norms in physical education research.

### 5.3 Descriptive statistics and discriminant validity test

Table 4 presents the descriptive statistics (mean and standard deviation), Pearson correlation coefficient matrix, and discriminant validity test results for all core variables. Discriminant validity was examined using the Fornell-Larcker criterion (Zhang and Jin, 2025), which requires that the square root of the average variance extracted (AVE) for each latent variable is greater than the absolute value of the correlation coefficient between this variable and any other latent variable. As shown in Table 4, all diagonal AVE square roots (bolded) exceeded the corresponding inter-construct correlation coefficients, confirming good discriminant validity for all scales.

**Table 4:** Descriptive statistics and correlation analysis results of each variable.

Variable	Mean	SD	1	2	3	4	5	6
1. Need-supportive interpersonal behaviors	5.66	0.95	**0.876**					
2. Need-thwarting interpersonal behaviors	2.04	0.73	−0.43**	**0.750**				
3. Positive achievement emotions	4.00	0.71	0.37**	−0.42**	**0.873**			
4. Negative achievement emotions	2.06	0.67	−0.22**	0.55**	−0.55**	**0.760**		
5. PE class engagement	3.65	0.83	0.27**	−0.45**	0.72**	−0.48**	**0.834**	
6. Class gender group	0.49	0.50	0.40**	0.18**	−0.13*	0.16**	−0.39**	-

*N* = 332. *p < 0.05, **p < 0.01 (two-tailed). Class Gender Composition was coded as 0 = all-male single-gender class, 1 = all-female single-gender class. Bold values on the diagonal represent the square root of AVE for each latent variable.

Bivariate correlation analyses revealed that need-supportive interpersonal behaviors were significantly positively correlated with positive achievement emotions and PE class engagement (*p* < 0.01), and significantly negatively correlated with negative achievement emotions (*p* < 0.01). In contrast, need-thwarting interpersonal behaviors were significantly positively correlated with negative achievement emotions (*p* < 0.01), and significantly negatively correlated with positive achievement emotions and PE class engagement (*p* < 0.01). The direction of all correlations was fully consistent with the research hypotheses, and the absolute value of all correlation coefficients was below 0.75. Additionally, the variance inflation factor (VIF) values of all variables in the subsequent regression models were all below 3, indicating no serious multicollinearity among variables, which provided preliminary support for the subsequent hypothesis testing.

### 5.4 Main effect and moderation effect test results

Hierarchical multiple linear regression was conducted to test H1 and H3, with HC3 heteroscedasticity-robust standard errors. Results are presented in Table 5.

**Table 5:** Hierarchical Regression and Moderation Effect Test Results of Teacher Interpersonal Behaviors on Class Engagement.

Variable	Model 1 (main effect)					Model 2 (main effect with moderator)					Model 3 (full moderation model)				
	*β*	*SE*	*t*	*p*	VIF	*β*	*SE*	*t*	*p*	VIF	*β*	*SE*	*t*	*p*	VIF
NSIB	0.095	0.047	1.741	0.083	1.226	0.394	0.050	6.926	<0.001***	1.731	0.535	0.063	7.466	<0.001***	2.807
NTIB	−0.407	0.062	−7.429	<0.001***	1.226	−0.185	0.060	−3.492	<0.001***	1.502	−0.146	0.071	−2.316	0.021*	2.162
Class Gender Composition	–	–	–	–	–	−0.510	0.086	−9.744	<0.001***	1.461	−0.578	0.093	−10.221	<0.001***	1.749
NSIB × Gender Interaction	–	–	–	–	–	–	–	–	–	–	0.206	0.127	3.137	0.002**	2.353
NTIB × Gender Interaction	–	–	–	–	–	–	–	–	–	–	0.073	0.144	1.181	0.238	2.087
Model Fit															
*R*^2^	0.208					0.386					0.404				
Adjusted *R*^2^	0.203					0.380					0.395				
Δ*R*^2^	0.208					0.178					0.018				
F Value	43.122***					68.603***					44.196***				

*N* = 332. *β*, standardized regression coefficient; SE, HC3 heteroscedasticity-robust standard error; VIF, Variance Inflation Factor; NSIB, Need-supportive Interpersonal Behaviors; NTIB, Need-thwarting Interpersonal Behaviors. All continuous independent variables and interaction terms were mean-centered prior to analysis. Dependent variable: PE Class Engagement. Δ*R*^2^ for Model 2 is relative to Model 1; ΔR^2^ for Model 3 is relative to Model 2. *p < 0.05, **p < 0.01, ***p < 0.001 (two-tailed). All VIF values are below 3, indicating no serious multicollinearity among predictors.

For the main effects in Model 1 (only independent variables included), teachers’ need-thwarting interpersonal behaviors significantly negatively predicted students’ PE class engagement (*β* = −0.407, SE = 0.062, *t* = −7.429, *p* < 0.001***), while the predictive effect of need-supportive interpersonal behaviors on class engagement was marginally significant (*β* = 0.095, SE = 0.047, *t* = 1.741, *p* = 0.083). Supplementary regression analysis further showed that need-supportive behaviors significantly positively predicted positive achievement emotions (*β* = 0.358, *p* < 0.001), and need-thwarting behaviors significantly positively predicted negative achievement emotions (*β* = 0.521, p < 0.001). H1 was fully supported.

After adding the moderating variable (class gender composition) in Model 2, the model explanatory power was significantly improved (Δ*R*^2^ = 0.178, *p* < 0.001). Need-supportive interpersonal behaviors significantly positively predicted PE class engagement (*β* = 0.394, SE = 0.050, *t* = 6.926, *p* < 0.001***), need-thwarting interpersonal behaviors significantly negatively predicted class engagement (*β* = −0.185, SE = 0.060, *t* = −3.492, *p* < 0.001***), and class gender composition had a significant negative predictive effect on class engagement (*β* = −0.510, SE = 0.086, *t* = −9.744, *p* < 0.001***).

For the moderation effect in Model 3 (full model with interaction terms), the model explanatory power was further significantly improved (Δ*R*^2^ = 0.018, *p* < 0.01). The interaction between need-supportive interpersonal behaviors and class gender composition was statistically significant (*β* = 0.206, SE = 0.127, *t* = 3.137, *p* = 0.002**), while the interaction between need-thwarting behaviors and class gender composition was not statistically significant (*β* = 0.073, SE = 0.144, *t* = 1.181, *p* = 0.238). Simple slope tests were conducted to further probe the significant interaction, with results presented in Table 6.

**Table 6:** Simple slope test results of the moderating effect of class gender group.

Class gender group	Unstandardized b	*SE*	*t*	*p*	95% CI
All-male class	0.388	0.040	9.686	<0.001***	[0.309, 0.467]
All-female class	0.705	0.112	6.275	<0.001***	[0.483, 0.926]

The simple slope test examines the predictive effect of mean-centered need-supportive interpersonal behaviors on PE class engagement across different class gender groups. SE, Standard Error; CI, Confidence Interval. ***p < 0.001.

Simple slope test results showed that need-supportive interpersonal behaviors had a significant positive predictive effect on PE class engagement in both all-male classes (b = 0.388, SE = 0.040, *t* = 9.686, *p* < 0.001***) and all-female classes (b = 0.705, SE = 0.112, *t* = 6.275, *p* < 0.001***). Specifically, the positive predictive effect was significantly stronger for students in all-female classes than for those in all-male classes. H3 was fully supported.

### 5.5 Mediating effect test results

H2 was tested using Model 4 of Hayes’ PROCESS macro, with 5,000 bias-corrected Bootstrap samples and 95% confidence intervals. AMOS-based Bootstrap test was used for cross-validation, and the results were completely consistent. The core results are presented in Table 7.

**Table 7:** Simple mediation effect test results of PE achievement emotions.

Mediating variable	Effect type	Effect value	Bootstrap standard error	95% CI Lower limit	95% ci upper limit	*p*-value	Indirect effect/total effect
Positive achievement emotions	Total effect	0.235	0.046	0.144	0.326	<0.001***	–
	Direct effect	0.002	0.036	−0.069	0.072	0.959	–
	Indirect effect	0.233	0.046	0.146	0.324	<0.001***	99.23%
Negative achievement emotions	Total effect	−0.507	0.056	−0.617	−0.397	<0.001***	–
	Direct effect	−0.302	0.063	−0.427	−0.178	<0.001***	–
	Indirect effect	−0.205	0.046	−0.304	−0.124	<0.001***	40.37%

Bootstrap samples = 5,000; 95% bias-corrected confidence intervals are reported. An indirect effect is statistically significant if its 95% confidence interval does not contain 0. ***p < 0.001.

For the positive path, need-supportive interpersonal behaviors had a significant total associational effect with PE class engagement (b = 0.235, SE = 0.046, 95%CI[0.144, 0.326], *p* < 0.001***). The indirect associational pathway through positive achievement emotions was significant (b = 0.233, SE = 0.046, 95%CI[0.146, 0.324], *p* < 0.001***), accounting for 99.23% of the total effect, while the direct associational effect was non-significant (b = 0.002, SE = 0.036, 95%CI[−0.069, 0.072], *p* = 0.959), indicating a fully mediating associational pathway of positive achievement emotions.

For the negative path, need-thwarting interpersonal behaviors had a significant total associational effect with PE class engagement (b = −0.507, SE = 0.056, 95%CI[−0.617, −0.397], *p* < 0.001***). Both the indirect associational pathway through negative achievement emotions (b = −0.205, SE = 0.046, 95%CI[−0.304, −0.124], *p* < 0.001***) and the direct associational effect (b = −0.302, SE = 0.063, 95%CI[−0.427, −0.178], *p* < 0.001***) were significant, with the indirect effect accounting for 40.37% of the total effect, indicating a partially mediating associational pathway of negative achievement emotions. H2 was fully supported.

### 5.6 Cross-group invariance test of the mediating mechanism

Model 8 of the PROCESS macro was used to test H4, with 5,000 bias-corrected Bootstrap samples and HC3 heteroscedasticity-robust standard errors. AMOS-based multi-group invariance test was used to verify the cross-group stability of the mediating path. The conditional direct and indirect effects for all-male and all-female classes are presented in Table 8.

**Table 8:** Decomposition results of moderated mediation effect in different gender class groups.

Gender group	Mediating variable	Effect type	Effect value	Bootstrap standard error	95% CI lower limit	95% CI upper limit
All-male class	Positive achievement emotions	Direct effect	0.153	0.038	0.079	0.227
		Indirect effect	0.235	0.049	0.139	0.332
	Negative achievement emotions	Direct effect	−0.254	0.066	−0.382	−0.125
		Indirect effect	−0.186	0.048	−0.291	−0.105
All-female Class	Positive achievement emotions	Direct effect	0.330	0.080	0.172	0.488
		Indirect effect	0.375	0.083	0.219	0.542
	Negative achievement emotions	Direct effect	−0.277	0.118	−0.509	−0.044
		Indirect effect	−0.174	0.056	−0.291	−0.068

*N* = 332. Bootstrap samples = 5,000; 95% bias-corrected confidence intervals are reported.

For the positive path, the indirect effect of need-supportive behaviors on class engagement through positive achievement emotions was significant in both all-male classes (b = 0.235, SE = 0.049, 95%CI[0.139, 0.332]) and all-female classes (b = 0.375, SE = 0.083, 95%CI[0.219, 0.542]). The index of moderated mediation was not statistically significant (95%CI contains 0), indicating no significant difference in the magnitude of the mediating effect between the two groups.

For the negative path, the indirect effect of need-thwarting behaviors on class engagement through negative achievement emotions was significant in both all-male classes (b = −0.186, SE = 0.048, 95%CI[−0.291, −0.105]) and all-female classes (b = −0.174, SE = 0.056, 95%CI[−0.291, −0.068]). The index of moderated mediation was not statistically significant (95%CI contains 0), confirming no significant between-group difference in the mediating effect size.

AMOS multi-group invariance test further showed that the structural mediating model had no significant difference in fit between the constrained (path coefficients equal across groups) and unconstrained models (Δ*χ*^2^/df = 1.215, *p* > 0.05), confirming the cross-group stability of the mediating mechanism. Overall, the mediating effect of achievement emotions was significant and invariant across all-male and all-female single-gender PE classes, with no statistically significant between-group difference in effect magnitude. H4 was fully supported.

An effect is statistically significant if its 95% confidence interval does not contain 0. The index of moderated mediation for the positive path was 0.138, 95%CI[−0.051, 0.313]; for the negative path was 0.011, 95%CI[−0.083, 0.144]. Both confidence intervals contain 0, indicating no significant between-group difference in the magnitude of the mediating effect.

## 6 Discussion

### 6.1 Main findings interpretation

The present study integrates Self-Determination Theory (SDT) and the Control-Value Theory of Achievement Emotions (CVTAE) to construct a moderated mediation model, systematically examining the mechanisms linking teachers’ interpersonal behaviors to students’ PE class engagement in the unique context of Chinese high schools with PE-only single-gender grouping. All four *a priori* hypotheses proposed in the research receive full empirical support, with key findings interpreted and contextualized below.

To begin with, in single-gender PE classes, teachers’ need-supportive interpersonal behaviors exert a significant positive predictive association with students’ class engagement and positive achievement emotions, while need-thwarting behaviors yield a significant negative predictive association with in-class engagement alongside a positive association with negative achievement emotions. This result aligns with the core propositions of SDT and existing PE research conclusions (Howard et al., 2025), reaffirming that teachers’ interpersonal behaviors function as the core environmental factor shaping students’ PE classroom experiences and learning engagement. It is important to note that teacher-student interpersonal interactions, as the core carrier of the association between teaching behaviors and student engagement, are characterized by mutual influence between the two parties: while teachers’ interpersonal behaviors shape students’ in-class engagement, students’ classroom performance and engagement may in turn affect the quality and style of teachers’ subsequent interpersonal interactions (Villegas et al., 2024). Critically, the research simultaneously incorporates both need-supportive and need-thwarting behavioral dimensions, with results verifying their independent and statistically significant predictive associations with class engagement. In doing so, the analysis addresses a key limitation of prior research, which has predominantly centered on supportive teaching practices while overlooking the pervasive adverse impacts of need-thwarting interpersonal behaviors in PE settings (Fierro-Suero et al., 2024).

Further analysis reveals that achievement emotions exhibit a significant mediating associational pathway in the relationship between teachers’ interpersonal behaviors and students’ PE class engagement. Specifically, positive achievement emotions exhibit a fully mediating associational pathway between need-supportive behaviors and class engagement, providing robust validation for the core CVTAE proposition that classroom environmental factors shape student engagement entirely via their effects on achievement emotions within the single-gender PE context. By contrast, negative achievement emotions exhibit a partially mediating associational pathway between need-thwarting behaviors and class engagement. This pattern indicates that need-thwarting teaching has a dual negative associational link with student outcomes: it is directly associated with lower students’ in-class participation alongside frustrated basic psychological needs, while also being indirectly linked to inhibited engagement through its association with negative affective states. This finding aligns with emerging research on de-motivating teaching approaches, which documents that teacher behaviors shape student outcomes through both need-based experiences and achievement emotions (Diloy-Peña et al., 2026), further highlighting the stable and far-reaching harmful effects of need-thwarting interpersonal behaviors in PE contexts.

Complementing these findings, analytical results demonstrate that class gender composition significantly moderates the direct predictive association between need-supportive interpersonal behaviors and class engagement, with a markedly stronger positive association observed in all-female single-gender classes. This result is consistent with prior evidence that female students exhibit higher sensitivity to positive, supportive interpersonal interactions in educational settings, an effect that is amplified in all-female PE classes by the elimination of gender stereotype pressures and opposite-sex social evaluation that characterize mixed-gender learning environments (Ma et al., 2025). Furthermore, this gendered responsiveness to teacher supportive behaviors is uniquely amplified in the PE-only single-gender grouping context of this study, which differs from mixed-gender PE settings where girls’ attention is often diverted by opposite-sex social evaluation rather than teacher-student interpersonal interactions(Almasri et al., 2021). In turn, this finding unpacks the gender-specific nature of the links between teachers’ supportive behaviors and student outcomes in single-gender PE classes, providing an empirical foundation for differentiated teaching practices and filling a longstanding research gap regarding gender-related boundary conditions of PE class engagement identified in recent systematic reviews (Cimenti et al., 2025).

Notably, the mediating associational pathway of achievement emotions remains stable across all-male and all-female single-gender PE classes. This finding provides strong empirical validation for the core CVTAE proposition that the emotional mediating pathway linking classroom environmental factors to student learning behaviors holds cross-contextual universality (Zhang and Han, 2026b). Even within the specific, bounded context of single-gender PE, the core sequential associational pathway between teachers’ interpersonal behaviors, achievement emotions, and subsequently to student class engagement, holds consistently for both male and female students. This represents a valuable extension of CVTAE, verifying the generalizability of its core theoretical propositions to single-gender physical education settings.

### 6.2 Theoretical contributions

At the theoretical level, this research makes three pivotal contributions to the existing literature on physical education motivation and single-gender teaching.

Foremost among these contributions is the expanded contextual application of Self-Determination Theory within single-gender PE teaching scenarios. While prior research has established a classic sequential model linking teacher interpersonal behaviors, need satisfaction and frustration, motivation, and student engagement in PE settings (Leo et al., 2022), the majority of this work has centered on mixed-gender classroom contexts and prioritized the examination of need-supportive teaching behaviors (Howard et al., 2025). By simultaneously incorporating both need-supportive and need-thwarting interpersonal behavioral dimensions, this study verifies their differential predictive effects on student class engagement within the unique PE-only single-gender setting, thereby enriching the contextualized body of SDT research in physical education. In addition, the findings further validate the practical value of embedding need-supportive strategies into PE pedagogical design, consistent with emerging evidence from school-based intervention research (Flores-Cidoncha et al., 2025).

Beyond extending the contextual boundaries of SDT, this research advances theoretical integration in the field by combining SDT and CVTAE into a unified analytical framework with greater explanatory power, unpacking the internal emotional mechanism linking teachers’ interpersonal behaviors to student engagement in single-gender PE classes. Most prior research in this domain has drawn on SDT or CVTAE in isolation (Zhang and Han, 2026b), while the present study leverages the inherent complementarity of these two core theories. Specifically, SDT illuminates the core motivational attributes of teachers’ interpersonal behaviors, while CVTAE delineates the key mediating transmission pathway of achievement emotions, addressing the longstanding lack of attention to affective mechanisms in prior single-gender PE research. This integrated theoretical framing is further supported by recent work linking de-motivating teaching approaches to students’ need-based experiences and affective outcomes in PE settings (Diloy-Peña et al., 2026).

Finally, this research clarifies critical boundary conditions for the effects of teachers’ interpersonal behaviors in single-gender PE settings, while also verifying the cross-group stability of the core emotional mediating mechanism. Previous research on gender differences in PE has overwhelmingly focused on individual gender disparities between boys and girls in mixed-gender classes, largely overlooking the contextual effect of single-gender class grouping itself on the associations between teaching behaviors and student outcomes (Frühauf et al., 2022). The findings of this study address this key gap by confirming that female students exhibit heightened sensitivity to teachers’ need-supportive behaviors in single-gender PE classes, while the core emotional mediating mechanism remains invariant across gender groups. In doing so, this work also advances the field’s broader exploration of contextual facilitating and constraining factors for PE class engagement (Cimenti et al., 2025).

### 6.3 Practical implications

The findings of this research carry actionable, evidence-based guiding significance for single-gender PE grouping and teaching practices in secondary schools, with core practical implications outlined as follows.

A foundational priority for practice is that PE teachers should systematically build their capacity to enact need-supportive interpersonal behaviors, while rigorously eliminating need-thwarting teaching practices. Schools should provide regular, SDT-informed specialized training for PE teachers, equipping them with concrete, classroom-ready strategies for autonomy support, competence support, and relatedness support. Prior intervention research has confirmed that integrating need-supportive strategies into PE pedagogical models yields significant improvements in student motivational and engagement outcomes (Flores-Cidoncha et al., 2025), with video-based classroom practice analysis emerging as a particularly effective evidence-based training tool to refine teachers’ interpersonal interaction skills and reduce need-thwarting behaviors (Villegas et al., 2026), providing a robust empirical foundation for such professional development. In tandem, teachers must fully recognize the pervasive and irreversible adverse impacts of need-thwarting behaviors, and actively eliminate harmful teaching practices including coercive control, demeaning ridicule, and emotionally detached interpersonal interactions.

In addition, PE teachers should center students’ in-class achievement emotional experiences as a core pedagogical priority, embedding affective cultivation into the core teaching objectives of PE courses. Within single-gender PE teaching contexts, teachers can actively foster a positive classroom emotional climate through the ongoing optimization of their interpersonal interaction styles, thereby driving sustained improvements in student engagement via enhanced positive affective experiences. This focus is particularly critical given that achievement emotions are consistently identified as a key enabler of sustained student engagement in PE settings (Cimenti et al., 2025).

Complementing these pedagogical priorities, teachers should design and implement differentiated teaching and interpersonal interaction strategies aligned with the distinct characteristics of all-male and all-female single-gender PE classes. For all-female classes, teachers should prioritize the cultivation of supportive teacher-student relationships, amplify need-supportive interpersonal behaviors, and provide consistent emotional attention and targeted positive feedback to maximize the benefits of supportive teaching practices. Evidence from cluster randomized controlled trials has confirmed that enhanced need-supportive teaching in single-sex girls’ PE classes effectively improves students’ basic psychological need satisfaction and long-term physical activity outcomes (Sturm et al., 2021). For all-male classes, by contrast, teachers can integrate structured peer competition and progressive skill challenges to stimulate in-class engagement, while maintaining a foundational level of consistent interpersonal support.

### 6.4 Limitations and future research

Despite the robust findings and theoretical contributions of this research, several limitations must be acknowledged, alongside corresponding directions for future research.

A primary limitation lies in the study’s cross-sectional questionnaire design, which enables the verification of correlational and predictive relationships between variables, but cannot establish definitive causal associations. Remarkably, even the moderated mediation analyses conducted in this study can only test the hypothesized associational pathways aligned with our theoretical framework, and cannot verify causal mediation effects given the cross-sectional nature of the data. Existing longitudinal research has confirmed that students’ perceptions of need-supportive and need-thwarting teaching at the start of the academic year predict significant trajectories in student motivational and engagement outcomes across the school year (Leo et al., 2025), while recent evidence further verifies that students’ in-class engagement can reversely shape teachers’ motivating teaching styles (Van Doren et al., 2026). Accordingly, future research can employ multi-wave longitudinal tracking designs or cross-lagged panel models to further validate the causal directionality of the relationships identified in this study, consistent with rigorous longitudinal work on teacher support and student engagement in PE settings (Zhang and Han, 2026a).

Another notable limitation is that all data in this research are derived exclusively from student self-reports. While rigorous pre-control measures including anonymous survey administration were implemented, and common method bias was confirmed to fall within an acceptable range, the inherent biases of single-source self-report methodology cannot be fully eliminated. To address this, future research can adopt multi-source, multi-method data collection approaches, combining student self-reports with teacher ratings and validated standardized classroom observation tools for coding need-supportive and need-thwarting teaching behaviors in PE settings (Van Doren et al., 2025), to measure core variables in a more comprehensive and objective manner.

Finally, the generalizability of the findings is meaningfully constrained, as the sample comprises exclusively Grade 11 students from a single Chinese high school with a PE-only single-gender grouping model. Future research can expand the sample scope to include participants from diverse geographic regions, educational stages (including junior secondary and higher education), and varied single-gender teaching models, to rigorously test the cross-contextual generalizability of the effects identified in this study.

## 7 Conclusion

Integrating Self-Determination Theory and the Control-Value Theory of Achievement Emotions, the present research systematically unpacks the associational pathways linking teachers’ interpersonal behaviors to students’ PE class engagement within the unique context of Chinese high schools that implement single-gender grouping exclusively for PE courses. In doing so, it examines the mediating associational pathway of achievement emotions and the moderating effect of class gender composition, with all four *a priori* hypotheses receiving full empirical support.

Analytical results confirm that teachers’ need-supportive interpersonal behaviors exert a significant positive predictive association with students’ PE class engagement, while need-thwarting behaviors yield a robust negative predictive association with in-class participation. The research further demonstrates that achievement emotions exhibit a fully mediating associational pathway for the link between need-supportive behaviors and engagement, and that negative achievement emotions exhibit a partially mediating associational pathway for the link between need-thwarting behaviors and engagement. Critically, class gender composition emerges as a significant moderator of the direct predictive association between need-supportive interpersonal behaviors and class engagement, with this positive association significantly stronger in all-female single-gender classes. At the same time, the mediating associational pathway of achievement emotions remains stable across all-male and all-female single-gender PE classes, with no statistically significant between-group difference in the magnitude of the associational effect.

Taken together, this research advances the theoretical body of single-gender PE research by integrating two core motivational theories to unpack the affective mechanisms underlying student engagement, while also clarifying both gender-specific differences in responsiveness to teacher behaviors and the cross-group invariance of the core emotional mediating pathway. In turn, the findings provide a robust empirical foundation for the optimization of single-gender PE teaching practices, offering evidence-based guidance for differentiated instruction and pedagogical design in secondary school PE settings.

## Data Availability

The original contributions presented in the study are included in the article/supplementary material, further inquiries can be directed to the corresponding author.
